# The Effect of Contact Lens–spectacle Reversed Galilean Telescope on the Visual Field of Patients with Open-angle Glaucoma

**DOI:** 10.18502/jovr.v15i4.7779

**Published:** 2020-10-25

**Authors:** Mohammadreza Moniritilaki, Maryam Badakhsh, Asieh Ehsaei, Ramin Daneshvar

**Affiliations:** ^1^Department of Optometry and Vision Science, University of Melbourne, Victoria, Australia; ^2^Refractive Errors Research Center, Mashhad University of Medical Sciences, Mashhad, Iran; ^3^Department of Optometry, School of Paramedical Sciences, Mashhad University of Medical Sciences, Mashhad, Iran; ^4^Eye Research Center, Mashhad University of Medical Science, Mashhad, Iran

**Keywords:** Glaucoma, Reversed Galilean Telescope, Visual Field

## Abstract

**Purpose:**

Glaucoma causes irreversible visual field defects. This study aims to evaluate the effect of a reversed Galilean telescope on the visual field of patients with open-angle glaucoma.

**Methods:**

Fifty-two glaucoma patients with a restricted visual field were recruited for this study. Central 30° visual field measurements were performed using a Humphrey visual field analyzer before and after applying the reversed Galilean telescope. To be more cosmetically acceptable, a combination of contact lens–spectacle was used as the reversed Galilean telescope.

**Results:**

Our data analysis showed that the reversed Galilean telescope had a significant effect on all measured perimetric indices. Visual field index (VFI) improved from a basic value of 44.38 ± 26.96 to 49.30 ± 29.83 percent by using the reversed telescope (*P* < 0.001). Moreover, the mean deviation (MD) was significantly improved from the initial value of –19.91 ± 7.19 dB to a value of –18.69 ± 7.73 dB (*P* < 0.001). However, our results showed a significant reduction in the pattern standard deviation (PSD) comparing before (9.83 ± 2.82) and after (8.51 ± 3.30) values using the reversed Galilean telescope (*P* < 0.001).

**Conclusion:**

The contact lens–spectacle combination reversed Galilean telescope significantly improved the central 30° visual field of glaucoma patients with the restricted visual field.

##  INTRODUCTION

Optical enhancement of restricted visual field in patients with visual field defects is a major concern of practitioners.^[1]^ Reversed telescopic lenses have been widely used as field expanding devices to help patients with severely constricted visual filed in low vision clinics.^[1,2,3]^

Visual field defect is a known, disabling consequence of many diseases such as glaucoma and retinitis pigmentosa (RP).^[1,4]^ Glaucoma is an irreversible, progressive optic neuropathy and its gradual visual field loss usually begins in the peripheral field. The peripheral visual field loss due to glaucoma causes decreased health-related quality of life.^[5]^ Epidemiological studies suggested that glaucoma causes bilateral blindness in nearly 6.7 million people worldwide. Based on these studies, there are approximately 67 million glaucomatous patients in the world.^[6]^

Although patients with the restricted visual field have high demands and motivations to use visual field expanding instruments, few devices are available.^[7]^ The reversed Galilean telescope is an optical system which has been used as visual field expander to help patients with constricted visual field due to various underlying etiologies.^[1,8,9]^

Mehr and Quillman^[8]^ reported a 63-year-old man who suffered from peripheral visual field loss as a result of RP and demonstrated a noticeable visual field improvement from 5° central to 14° after using the reversed Galilean telescope. Their patient also reported that using the reversed Galilean telescope was very useful in his daily tasks and had an unexpected social advantage for him. In a larger series, Kennedy et al^[1]^ assessed 10 participants with severe visual field loss due to RP. They evaluated visual acuity and visual field before and after using the reversed Galilean telescope and reported subjective improvement in the visual field of six subjects. Both of these studies have shown visual field improvement after using the reversed Galilean telescope in participants who suffered peripheral visual field loss due to RP.

In this study, we evaluated the hypothesis that using a reversed Galilean telescope can improve the visual field of advanced glaucoma cases. We also used a modified version of the reversed Galilean telescope which is cosmetically more acceptable. Additionally, we evaluated the visual field with Humphrey field analyzer which is more accurate than previously used tangent screen.

##  METHODS

### Participants

Fifty-two participants between the ages of 18 and 73 years (mean age 53.8 ± 13.0 years; 12 (24%) female) were recruited for this study. Comprehensive eye examinations were performed to determine their eligibility. All included subjects had advanced primary open-angle glaucoma (based on the Hodapp-Anderson-Parrish (HAP) criteria), a best-corrected visual acuity of 20/200 or better, and at least three reliable visual fields on their records. An absolute spherical refractive error of fewer than 4.00 dioptres (D) with < 0.50 D of the cylinder was also an inclusion criterion. Prior to the experiment, all participants provided a written informed consent. The study protocol was approved by the Research Ethics Committee of Mashhad University of Medical Sciences and adhered to the tenets of the Declaration of Helsinki.

#### Field expanding device

Technically, field expansion could be achieved by using a special optical system to minify the image size. The reversed Galilean telescope is one of the most useful devices which has been used for over three decades in many studies to expand the visual field.^[1,2,3]^ It consists of one converging lens serving as the eyepiece and a diverging lens as the objective.

Although many studies have shown improvement in the restricted visual field after using the reversed Galilean telescope,^[1,8,9]^ it is cosmetically not acceptable for most patients. In this study, we used an innovative, modified model of the reversed Galilean telescope to be more user-friendly and practical. We used a contact lens as the eyepiece and a spectacle with a negative lens as the objective part of the telescope.

To create a reversed Galilean telescope, we used a spectacle with a constant power of –2.00 D for all participants. To calculate the power of the contact lens (Bausch & Lomb, Rochester, New York, USA), we first measured the refraction for the far glasses and the dioptric addition they needed for near correction to do the perimetry. Then, +2.00 D was added to the sum of far and near refractions. For example, in a patient with a far spectacle of +1.50 D and a near addition of +2.00 D, we put a +5.50 D contact lens over the cornea and a –2.00 D corrective lens in the lens holder of Humphrey perimeter to create a reverse Galilean telescope.

#### Visual field measurement

Visual field measurement was performed with a Humphrey visual field analyzer (HFA-II, Carl-Zeiss Meditec, Dublin, CA, USA) using the Swedish Interactive Threshold Algorithm (SITA)- standard strategy and the central 30-2 program. We measured three global parameters of the HFA, namely the visual field index (VFI), mean deviation (MD), and pattern standard deviation (PSD), to assess the visual field changes after using the reversed Galilean telescope.^[10,11,12,13,14]^ Visual field measurement was performed on the eligible eyes. If both eyes of a participant met the inclusion criteria, the eye with the worse visual field defect was included in the study.

#### Study protocol

The following protocol was used for all participants. First, comprehensive ophthalmologic examinations were performed by a glaucoma fellowship-trained ophthalmologist to determine the eligibility of participants and the refractive status was evaluated by an experienced optometrist. Before the main visual field evaluation in the experimental session, 5 min run was performed to ensure that the participants were familiar with the visual field test procedure. All included subjects had two successive visual field measurements with and without the introduction of the reversed Galilean telescope and the order of the tests was randomized. There was a 1-hour rest period between the two tests.

#### Statistical analysis

All statistical analyses were performed using the Graphpad Prism 6 software (www.graphpad.com, San Diego, California, United States). Paired *t*-tests were conducted to compare the global indices before and after applying the reversed Galilean telescope.

##  RESULTS

**Figure 1:**
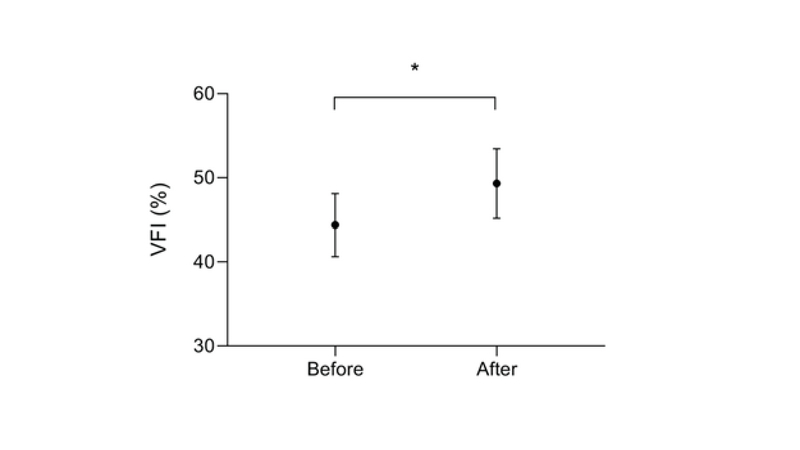
VFI changes before and after using the reversed Galilean telescope. Data are presented as Mean ± SEM (*n* = 52 subjects). *Denotes a significant difference.

**Figure 2:**
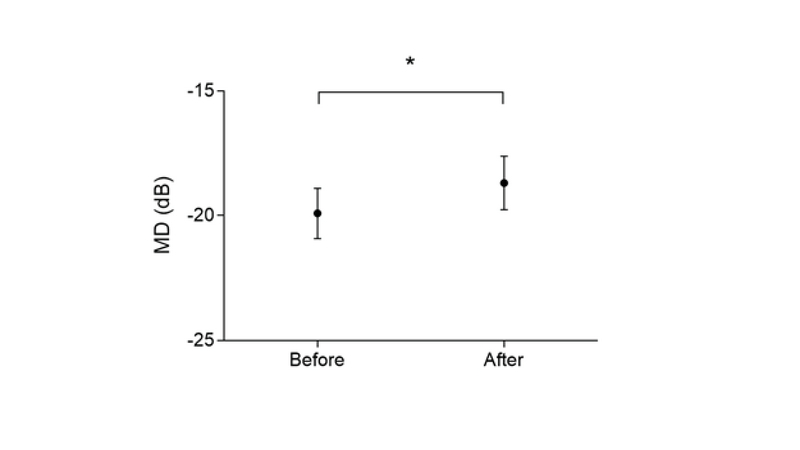
MD changes before and after using the reversed Galilean telescope. Data are presented as Mean ± SEM (*n* = 52 subjects). *Denotes a significant difference.

The mean refractive error (spherical equivalent) of participants was +1.17 ± 1.68 D, and the mean best-corrected visual acuity was 0.21 ± 0.25 LogMAR. All measured perimetric indices were significantly different after using the reversed Galilean telescope. Interestingly, VFI increased significantly from 44.38 ± 26.96 percent to 49.30 ± 29.83 percent by using the reversed telescope (*P* < 0.001) (Figure 1). Similarly, MD change was statistically significant from an initial value of –19.91 ± 7.19 dB to a post-intervention value of –18.69 ± 7.73 dB (*P* < 0.001) (Figure 2). Although the sensitivity of the retinal points had not been changed, the minified image transferred more test points from the damaged peripheral zone to the central, less damaged zone which resulted in a significant increase of MD and VFI.

**Figure 3:**
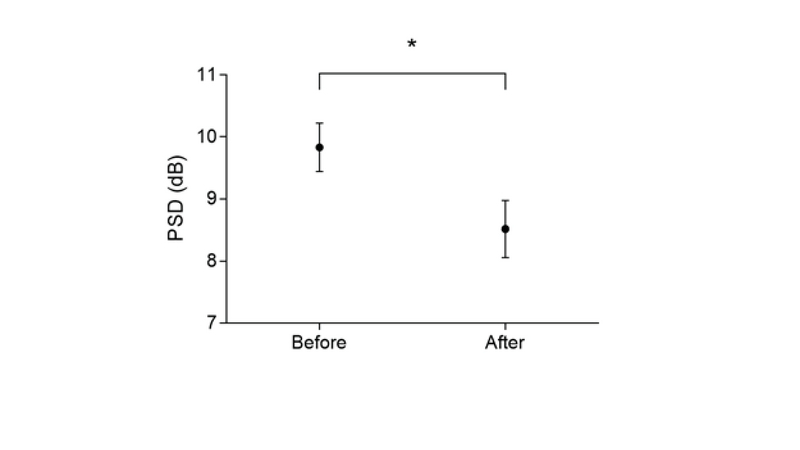
PSD changes before and after using the reversed Galilean telescope. Data are presented as Mean ± SEM (*n* = 52 subjects). *Denotes a significant difference.

Figure 3 illustrates the PSD changes before and after using the reversed Galilean telescope in the central 30°. Data analysis showed significant changes between the before (9.83 ± 2.82) and after (8.51 ± 3.30) values using the reversed Galilean telescope (*p* < 0.001).

##  DISCUSSION

In this study, we found that using the reversed Galilean telescope leads to a visual field improvement in central 30° of glaucomatous participants. We used the standard automated perimetry to quantify visual field changes after the administration of this device. This method leads to significant increments in the VFI and MD, denoting visual filed improvement. We also observed a significant decrement in the PSD index after using the telescope indicating increased homogeneity in the visual field. As the results of the VFI and MD suggest visual field improvement, the increase of homogeneity of the visual field has been interpreted as visual field enhancement.

Glaucoma patients usually have visual complaints more than what is estimated by a visual acuity eye chart.^[15]^ Moreover, glaucoma affects the ability to detect objects in low illumination and discriminating low-contrast objects which have been found important in daily activity.^[16]^ Visual field loss is a major disabling consequence of glaucoma and may cause difficulties in daily life.^[17]^ It has been demonstrated that even mild visual field defects can lead to a difficulty in outdoor navigation, which in turn results in anxiety and stress for the patients.^[18]^

Although, visual field defects are not reversible by current surgical and non-surgical treatments,^[19]^ using optical methods and minified images can potentially improve the visual field. Technically, these minifying optical systems bring images from peripherally damaged visual field to the central less damaged field, which result in improved visual field.^[20]^

This study showed that a reversed Galilean telescope caused a significant improvement in the visual field. All measured perimetric indices were significantly affected by the implemented reversed Galilean telescope. The use of this optical device resulted in a significant increment in MD and VFI and also significant reduction in PSD, expressing the visual field enhancement, which is consistent with the results of a study by Campbell et al^[9]^ who demonstrated a significant visual field enhancement from 4° central to 20–40° by using the reversed Galilean telescope. They reported that this optical system led to visual field improvement in a 25-year-old female who suffered from severe visual field constriction due to stroke.

Moreover, visual field improvement has been reported after using the reversed Galilean telescope in patients with restricted visual field due to RP.^[1,8]^ Kennedy and associates^[1]^ used an optical device as a reversed Galilean telescope consisting of three small lenses placed within a plastic tubular casing. The two front lenses which stick together formed the objective lens and the third one was the ocular lens. In another study, Mehr and Quillman^[8]^ showed a significant visual field enhancement after using the reversed Galilean telescope. They mounted a concave lens on the participant's spectacle which had a convex lens to create 1.3× reversed Galilean telescope. The reversed Galilean telescopes used in each of these studies were not cosmetically acceptable for patients. However, in the current study, a modified contact lens–spectacle combination was used as a reversed Galilean telescope which was more practical. The ocular piece of our device is a convex contact lens which is the result of adding +2.00 D to the summation of refraction and near addition. In addition, the objective lens in our optical system is a constant lens of –2.00 D which is placed in the lens holder of the Humphrey perimeter.

It is well-accepted that the retinal image size of a spectacle differs from a contact lens with the same power.^[21]^ In our telescope, because of farther distance to the nodal point, the minifying effect of –2.00 D spectacle is more than the magnifying effect of the +2.00 D contact lens. Therefore, the optical system causes a minified retinal image, resulting in a wider visual field.

In the standard automated perimeter, PSD reveals the localized visual field defect. A high PSD suggests more difference between the more and the less sensitive visual field points and a low PSD means there is either a homogenous defect across the visual field or there is no defect.^[14]^ Therefore, regarding the results of the MD and the VFI (showing improved visual field), decrement in PSD suggests improved visual field homogeneity which is compatible with the results of MD and VFI.

Various optical systems have been used over the decades to help patients with vision problems. Implanted miniature telescope is an example of a magnifying optical system that has been used to help patients with age-related macular degeneration (ARMD).^[22]^ This implantable optical system magnifies images, therefore bringing the image from the central visual field, which is damaged, to the peripheral visual field, leading to an increase in visual acuity and improved quality of life. Hudson and coworkers^[23]^ reported that the implantable miniature telescope in patients with end-stage ARMD causes three lines or more improvement in the best-corrected distance and near visual acuity and quality of life. On the other hand, the minifying optical system that has been used in this study displaces the image throughout the visual field. Unlike the mentioned implantable miniature telescope, the reverse Galilean telescope brings the image from the peripheral visual field to the central visual field which is less damaged; hence, resulting in the improved visual field.

To the best of our knowledge, this is the first report on using a reversed Galilean telescope to enhance the visual field of glaucoma patients; moreover, our particular contact lens–spectacle combination as a reversed Galilean telescope has not been previously reported in clinical practice. This approach has its limitations, including but not limited to difficulties in using a contact lens in elderly glaucomatous patients. Dry eye is a common co-morbidity in these patients and contact lens use may have more side effects. However, considering its proven efficacy, one can use this approach by proper case selection and patient education. In addition, a potential approach in treating the glaucomatous patient with cataract may make them somewhat myopic by implanting an over-powered intraocular lens. Then, a reversed Galilean telescope effect can be induced by using a corrective, minus spectacle to enhance the visual field. This is the subject of the authors' ongoing research.

##  Financial Support and Sponsorship

This study has received support from the Vice-Chancellor of Research, Mashhad University of Medical Sciences, Grant No. 910712.

##  Conflicts of Interest

There are no conflicts of interest.
